# Secretome of Olfactory Mucosa Mesenchymal Stem Cell, a Multiple Potential Stem Cell

**DOI:** 10.1155/2016/1243659

**Published:** 2016-02-01

**Authors:** Lite Ge, Miao Jiang, Da Duan, Zijun Wang, Linyu Qi, Xiaohua Teng, Zhenyu Zhao, Lei Wang, Yi Zhuo, Ping Chen, Xijing He, Ming Lu

**Affiliations:** ^1^Key Laboratory of Protein Chemistry and Developmental Biology of Ministry of Education, College of Life Sciences, Hunan Normal University, Changsha 410081, China; ^2^Department of Neurosurgery, Second Affiliated Hospital of Hunan Normal University (163 Hospital of PLA), Changsha 410003, China; ^3^Orthopedics Department, Second Affiliated Hospital, College of Medicine, Xi'an Jiaotong University, Xi'an 710004, China

## Abstract

Nasal olfactory mucosa mesenchymal stem cells (OM-MSCs) have the ability to promote regeneration in the nervous system* in vivo*. Moreover, with view to the potential for clinical application, OM-MSCs have the advantage of being easily accessible from patients and transplantable in an autologous manner, thus eliminating immune rejection and contentious ethical issues. So far, most studies have been focused on the role of OM-MSCs in central nervous system replacement. However, the secreted proteomics of OM-MSCs have not been reported yet. Here, proteins secreted by OM-MSCs cultured in serum-free conditions were separated on SDS-PAGE and identified by LC-MS/MS. As a result, a total of 274 secreted proteins were identified. These molecules are known to be important in neurotrophy, angiogenesis, cell growth, differentiation, and apoptosis, and inflammation which were highly correlated with the repair of central nervous system. The proteomic profiling of the OM-MSCs secretome might provide new insights into their nature in the neural recovery. However, proteomic analysis for clinical biomarkers of OM-MSCs needs to be further studied.

## 1. Introduction

Repairing the central nervous system (CNS) has always been a challenge of science prompting innovative strategies. There is an old view that the nerves have little potential to rebirth according to cognitive demands of the environment [1]. Even though neural stem cells (NSCs) were capable of self-renewal and generating the main phenotypes (neurons, astrocytes, and oligodendrocytes) of CNS cells* in vitro* and* in vivo* [2], endogenous NSCs usually fail to remedy the deleterious consequences of severe trauma or neurodegenerative diseases [3–5]. Therefore, exogenous cell therapy has been proposed as a promising approach to treat a variety of neurological diseases [6].

Among the candidate cells for clinical cell therapy, mesenchymal stem cells (MSCs) are considered to be an ideal cell type for nerve repair and clinical applications [7, 8]. MSCs could be obtained from a variety of tissues, including bone marrow, umbilical cord, umbilical cord blood, adipose tissue, synovium, skeletal muscle, and deciduous teeth [9]. Among them, bone marrow mesenchymal stem cells are well-studied. However, it is still faced with many restrictions, including high susceptibility to viral exposure, the invasiveness of marrow collection, the rarity of MSCs in the marrow, cell proliferation ability, and cell differentiation capacity decrease with aging [10–12]. Thus, researchers have never stopped looking for new seed cells for clinical cell transplantation.

Recently, nasal olfactory mucosa mesenchymal stem cells (OM-MSCs) stand as a promising competitor for therapeutic application [13–15]. It has the advantages of easy accession, advantageous localization, and high versatility [16]. Most importantly, OM-MSCs could be used for autologous transplantation. Presently, these cells have been successfully used in different mammals models, including myocardial infarct [17], spinal cord trauma [18–20], hippocampal lesions [21], Parkinson's disease [22], and cochlear damage [23, 24]. Although OM-MSCs present the advantage of potential usefulness for autologous transplantation, the detailed mechanisms are only partially understood.

It has been well-accepted that the reparative effect of OM-MSCs is due to not only its differentiation capacity, but also the paracrine factors [25, 26]. These paracrine factors might play important roles in creating a supportive microenvironment for cell survival and differentiation, the activation of endogenous NSCs, reduction of inflammatory reaction, and inducement of angiogenesis [27]. Therefore the secretome of OM-MSCs could be very useful to uncover the active ingredients for further study on its mechanism and clinical application.

Here, the secretome of OM-MSCs was investigated by SDS-PAGE and LC-MS. A total of 274 secreted proteins were identified. These molecules are known to be important in neurotrophy, angiogenesis, cell growth, differentiation, and apoptosis, and inflammation indicating that OM-MSC transplantation could significantly improve the repair of CNS. The proteins presented in this study represent the first secretome profiling of OM-MSC, which may provide insight into its functions in nerve repairment.

## 2. Materials and Methods

### 2.1. Ethics Statement

Human nasal mucosa biopsies were collected from 4 individuals, 2 males and 2 females aged 24 to 49 years, at the Second Affiliated Hospital of Hunan Normal University (Changsha, China); investigations were approved by the ethical committee of Hunan Normal University, China. Informed consent was given by each individual participating in the study, in accordance with the Helsinki convention (1964). Inferior turbinate tissues were discarded from 4 patients undergoing septoplasty/polypectomy surgery. The patients with history of facial trauma were excluded.

### 2.2. Tissue Culture

Following published protocol for the cultivation of human nasal lamina propria MSCs [16], samples were collected at the root of the medial aspect of the middle turbinate, washed 3 times at room temperature with antibiotic-antimycotic solution (Invitrogen, Carlsbad, CA), and then cut into 1 mm^3^ to 2 mm^3^ pieces with a thickness ranging from 200 to 500 *μ*m. The pieces were placed into a culture dish that was covered with a sterilized glass cover slide. Dulbecco's modified Eagle's medium (DMEM; Invitrogen) containing 10% fetal bovine serum (FBS) was added, and the tissues were incubated at 37°C in 5% CO_2_. The culture medium was changed every 2 or 4 days. Seven to eight days after, stem cells will begin to invade the culture dish and after 2-3 weeks they should be confluent. When confluency is reached, passage and transfer the cells to culture flasks. When almost confluent, cells were again harvested and were then plated in sphere-forming conditions. Cell density appeared to be important for robust sphere formation. Cells were plated at 4 × 10^5^ to 6 × 10^5^ cells per poly-L-lysine coated T25 flask in DMEM/F12 supplemented with 50 ng/mL fibroblast growth factor 2 (R&D Systems, Minneapolis, MN) and 50 ng/mL epidermal growth factor (R&D Systems). Medium was added every 2 to 4 days. Loosely adherent spheres were harvested by trituration and brief Accutase (Invitrogen) treatment for immunochemical analysis.

### 2.3. Immunofluorescent Staining

Immunofluorescence was carried out to analyze nestin, STRO-1, and microtubule-associated protein-2 (MAP-2) expression using standard protocols. After fixation and washing, cultures were blocked with 10% normal goat serum in 0.3% Triton X-100 for 1 hr at room temperature and incubated with the primary antibody at 4°C overnight. The following primary antibodies were used: monoclonal rabbit anti-nestin (1 : 250, Abcam, Cambridge, UK) and mouse anti-STRO-1 (1 : 250, Sigma-Aldrich, CA, USA) for OM-MSCs and monoclonal anti-MAP-2 (1 : 250, Abcam) for neuron-like cells. Then cultures were incubated with the appropriate conjugated secondary antibody for 1 hr, at room temperature in dark conditions. The following secondary antibodies were used: goat anti-mouse IgG conjugated with AlexaFluor 488 (1 : 500, Santa Cruz, CA, USA) and donkey anti-rabbit IgG conjugated with AlexaFluor 647 (1 : 500, Santa Cruz). After several washes in PBS, cultures were counterstained with DAPI (4′,6-diamidino-2-phenyl-indole; Roche, Germany) for 5 min and mounted with an antifading medium (Beyotime, Hangzhou, China). Images were taken with a fluorescence microscopy (Leica DM 2000, Germany).

### 2.4. Flow Cytometry Analysis

For surface protein expression, OM-MSCs were plated into a test tube (Becton Dickinson, NJ, USA) at a density of 1 × 10^5^/mL and washed three times with wash buffer (0.1% FBS/PBS). The cells were incubated for 40 min with saturating concentrations of fluorescent-conjugated monoclonal antibodies CD34, CD45, CD73, CD90, and CD105. Anti-CD34 was conjugated to ECD, anti-CD45 was conjugated to PC7, anti-CD73 and anti-CD90 were conjugated to FITC, and anti-CD105 was conjugated to PE. After washing, cell fluorescence signals were determined immediately using flow cytometry with a FACS Caliber instrument (Becton Dickinson, CA, USA). The analysis was performed using Cell Quest Software (Becton Dickinson, CA, USA) [28, 29].

### 2.5. Osteogenic and Adipogenic Differentiation

The ability of OM-MSCs to differentiate into adipogenic and osteogenic lineages was assayed on cells plated at 10^2^ to 10^3^ cells/cm^2^ in DMEM/F12, 10% FBS, and penicillin/streptomycin in 6-well plates. 24 hours later, using StemPro osteogenesis and adipogenesis differentiation kits (Invitrogen), induction factors were added to 3 wells and 3 wells kept as controls. Cells were fed every other day for 14 days and bone and fat were detected using alizarin red S stain, alkaline phosphatase stain, and Oil red O stain.

### 2.6. Neurogenic Differentiation

To induce neurogenesis, single OM-MSCs were placed in nonadherent 6-well culture plates with a density of 100,000 cells per mL for 24–48 hours in the DMEM/F12 medium supplemented with 10% FBS, penicillin/streptomycin. Then, the medium was replaced with DMEM/F12 supplemented with 500 nM retinoic acid (Sigma-Aldrich) for 12 days. The differentiated cells were used for western blot and immunocytochemistry of immature to mature neural markers such as Neuronal Class III *β*-tubulin-1 (*β*-TUB), MAP-2, nestin, and glial fibrillary acidic protein (GFAP).

### 2.7. Protein Extraction

Proteins of OM-MSCs were harvested by washing cultured and acutely dissociated OM-MSCs in cold PBS and lysing with 500 *μ*L of 1x SDS-PAGE sample buffer. Samples were incubated on ice and stored at −20°C.

To obtain secreted protein, approximately 80% confluent OM-MSC cultures were rinsed three times with PBS and incubated overnight in DMEM/F12 conditioned medium. The conditioned medium was collected and centrifuged at 500 g. The supernatant was filtered using a 0.2 *μ*m filter and placed in dialysis cassettes with molecular weight cut-off of 3500 (Pierce), dialyzed against three changes of 10 volumes of 1 mM ammonium bicarbonate solution. The dialyzed CM was lyophilized overnight and then resuspended in 1x SDS sample buffer.

Total proteins were quantified using the Bradford method.

### 2.8. SDS-PAGE and Western Blotting

SDS-PAGE protein gels were performed according to standard procedures. Equal amount (100 *μ*g) of proteins was boiled for 5 min and centrifuged at 12,000 g for 10 min at 4°C. Samples were subjected to SDS-PAGE using 11.5% separation gel and 4.8% stacking gel. After electrophoresis, the gel was stained using coomassie brilliant blue. To prepare samples for western blotting, 20–50 *μ*g samples were separated on SDS-PAGE, transferred to PVDF membrane (Millipore, MA, USA), and then probed with various antibodies overnight at 4°C. The dilution rates of primary antibodies were as follows: anti-*β*-TUB (1 : 200), anti-GFAP (1 : 400), anti-MAP-2 (1 : 400), and anti-nestin (1 : 300). Next, the membrane was incubated with HRP conjugated anti-rabbit or anti-mouse IgG, and after fully washing with TBS the blot was visualized using ChemiDoc XRS imaging system (Bio-Rad, CA, USA).

### 2.9. Tryptic Digestion and MS/MS Analysis

The stained gel was sliced into fifteen bands of equal size, and a standard protocol was performed for in-gel digestion as described before [30, 31]. The trypsinized sample was lyophilized, dissolved in 0.1% formic acid, and analyzed using an LTQ mass spectrometer (Thermo Finnigan, San Jose, CA) coupled with an Agilent 1200 capillary system (Agilent, Waldbronn, Germany). Mobile phase A (0.1% formic acid in H_2_O) and mobile phase B (0.1% formic acid in acetonitrile) were selected. A linear 65 min gradient was achieved from 3% to 60% B at a flow rate of 0.3 *μ*L/min. The eluted peptide ions were then detected by the LTQ mass spectrometer equipped with the nano-ESI source. The electrospray voltage was 1.9 kV, and 35% normalized collision energy was used for MS/MS. Data-dependent MS/MS spectra were obtained in which the five most abundant spectra from the full MS scan were selected for fragmentation. The following are the dynamic exclusion settings: the repeat count was set to 1, the repeat duration was 30 s, the dynamic exclusion duration was set to 180 s, the exclusion mass width was 1.5 Da, and the list of dynamic exclusion was 50.

### 2.10. Database Searching and Bioinformatics Analysis

Raw MS data were processed by the TurboSEQUEST program in the Bioworks Browser software suite (version 3.1, Thermo Electron, San Jose, CA). The following SEQUEST search parameters were used: precursor mass tolerance was set at 15 ppm and 0.8 Da for MS/MS fragments. The matched peptides were allowed up to 1 missed cleavage and oxidation on Met (+16 Da) as variable modification while carbamidomethylation on Cys (+57 Da) as fixed modification. Peptides whose ions scores exceeded the threshold (*p* < 0.05, 95% confidence level) were selected and a list of proteins that had a ProteinProphet probability greater than 0.99 and also had more than one unique peptide were obtained.

The accession number of each identified protein was loaded to the gene ontology (GO) classification system (http://www.geneontology.org/). The proteins were classified into the different cellular component, molecular function, and biological process. General functions analysis was performed with the tools on the Swiss-Prot database (http://www.uniprot.org/), respectively.

## 3. Results

### 3.1. Isolation of Human Olfactory Mucosa (OM) Biopsies

**Figure 1:**
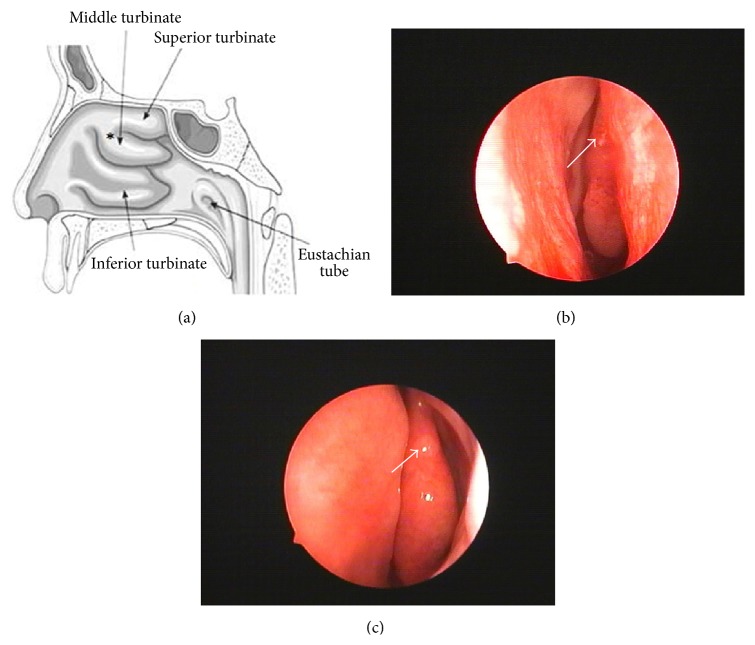
Otolaryngologic image of the nasal cavity. Schematic of the right nasal cavity, *∗*: at the root of the middle turbinate (a), endoscopic visualization of the obtaining (b), and endoscopic pictures after one month (c).

Before collection, certain diseases, especially nasal polyps and/or active sinus infection, are necessary to be excluded. The protocol was designed according to IRB after passing the ethical committee and all volunteers signed the informed consent for the use of intranasal biopsy samples for research. Then tissue samples were obtained from the root of the medial aspect of the middle turbinate undergoing endoscopic nasal surgery (Figure 1(a)). Usually OM of patients could heal within a month after injury. Moreover, there is no effect on the patients' sense of smell through olfactory measurement (Figures 1(b) and 1(c)).

### 3.2. Characterization and Sphere-Forming Capabilities of OM-MSCs

**Figure 2:**
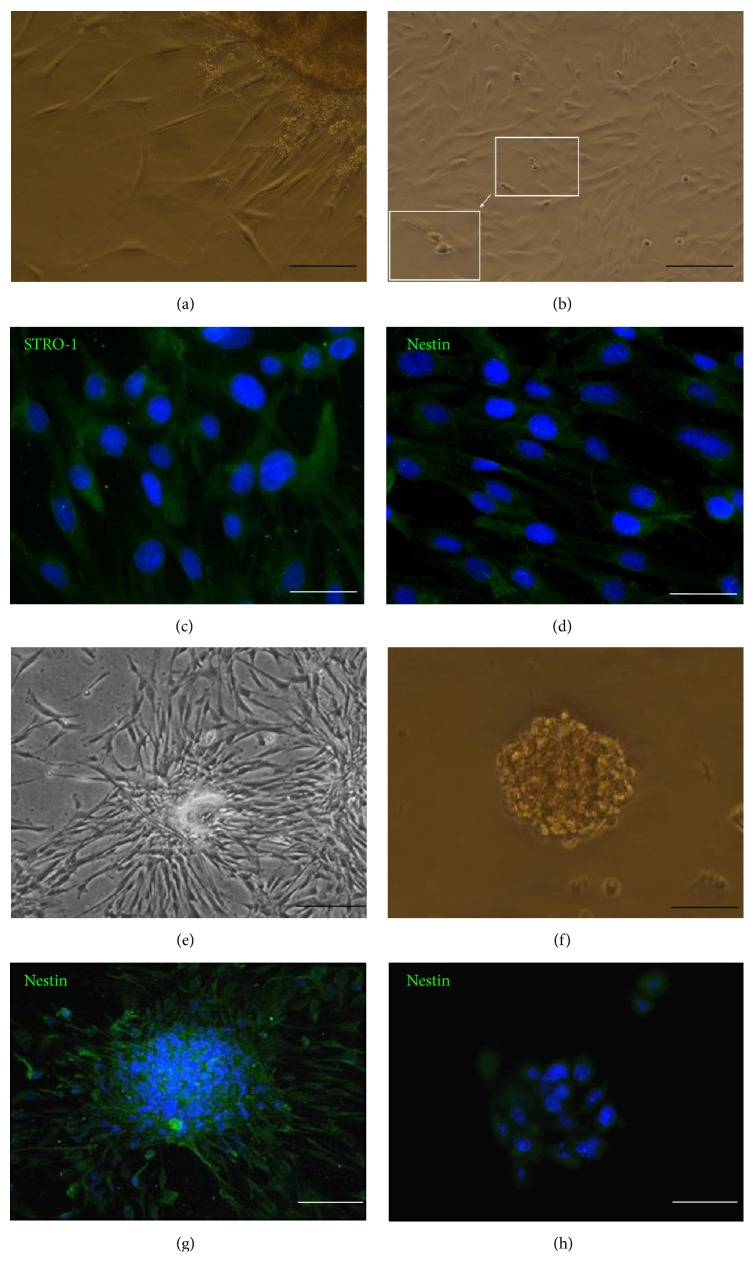
Characterization and sphere-forming capabilities of OM-MSCs. Adherent cells migrated from the explants and most cells became spindle shaped (a), the rapidly grown cells within nuclear disintegration and cell cleavage (b), immunocytochemistry of the characteristic markers of human OM-MSCs: STRO-1 ((c) and (g)) and nestin ((d) and (h)). After cultivating in the appropriate culture conditions, as expected, cells tended to aggregate; many loosely attached or floating spheres appeared ((e) and (f)). Neurospheres can be expressed as nestin throughout the whole process; nuclei are counterstained with DAPI (blue). Scale bars: (a) 100 *μ*m; (b) 200 *μ*m, white box: 100 *μ*m; (c) and (d) 50 *μ*m; (e) and (g) 200 *μ*m; (f) and (h) 50 *μ*m. DAPI = 4′,6-diamidino-2-phenylindole; MSC = mesenchymal stem cell.

To validate that the correct population of cells had been harvested from OM, we evaluated whether it was possible to isolate stem cells. Following the protocols for the generation of OM-MSC cultures, we successfully derived cells exhibiting a mesenchymal stem cell like morphology (Figures 2(a) and 2(b)). After 6 to 8 days in culture, adherent cells migrated from the explants and most cells became spindle shaped (Figure 2(a)). After passaging, the cells grow rapidly and the nuclear disintegration and cell division could be observed (Figure 2(b)), showing active proliferation capacity. Moreover, purified cells were immunopositive for STRO-1 and nestin, which are the characteristic markers of human OM-MSCs (Figures 2(c) and 2(d)) [14]. After culturing for 14 days, as expected, cells tended to aggregate to loosely attached or floating spheres (Figures 2(c) and 2(d)) in which the signal of nestin is strong (Figures 2(e) and 2(f)). Indeed, even though derived from very small pieces of tissue, millions of cells could be obtained within a month easily.

### 3.3. Surface Marker Expression

**Figure 3:**
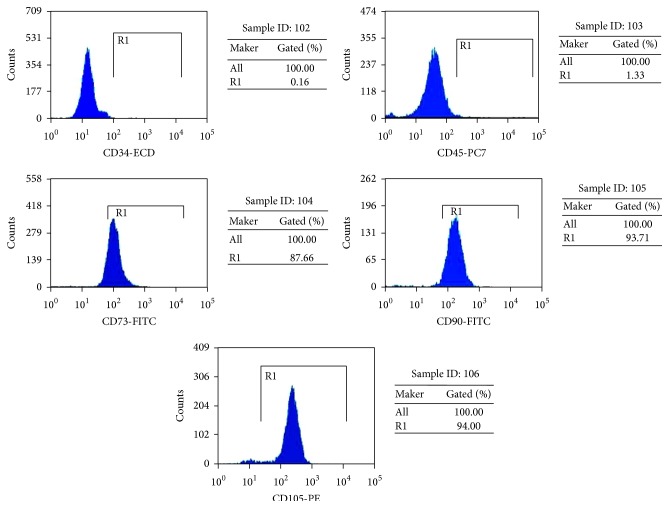
Surface marker expression. Flow cytometric analysis of these cells showed that they express the MSC markers CD73, CD90, and CD105, but not CD34 and CD45, which are characteristic of hematopoietic cells.

To determine the immunophenotyping profile of OM-MSCs, we used flow cytometry to assess different cell surface antigens. Flow cytometric analysis of these cells showed that they express the MSC markers CD73, CD90, and CD105, but not hematopoietic cells markers CD34 and CD45 (Figure 3). These results suggested that these cells are mainly composed of MSCs.

### 3.4. OM-MSCs Differentiate into Osteoblasts, Adipocytes, and Neurons* In Vitro*

Researches have shown that MSCs could differentiate into bone, fat, cartilage, and other types of cells; thus the pluripotency of the OM-MSCs was measured.

#### 3.4.1. *In Vitro* Adipogenic Assay

**Figure 4:**
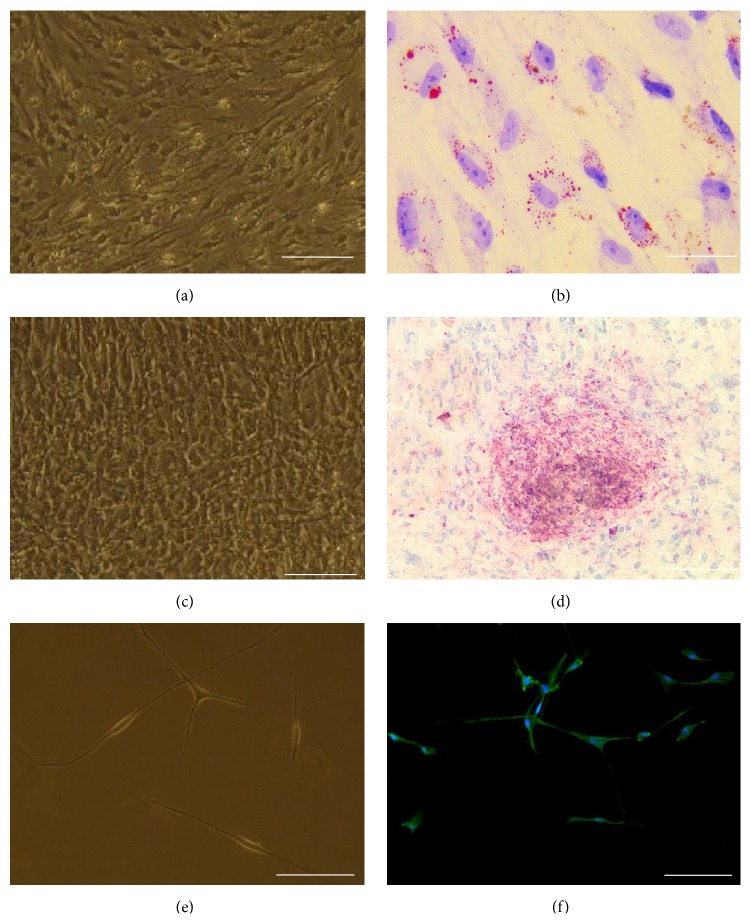
OM-MSCs differentiate* in vitro* into osteoblasts, adipocytes, and neurons. Adipogenic, osteogenic, and neuron differentiation was observed and assessed by Oil red O (ORO), alizarin red staining (ARS), or MAP-2 in induction media. (a) Adipocyte cellular morphology in 2 weeks; (b) lipid droplets that showed red color after ORO staining; (c) osteocyte cellular morphology in 3 weeks; (d) ARS that verified the formation of large calcium deposits. (e) Neuron cellular morphology in 12 days; (f) neuron-like cells that can be expressed MAP-2. Nuclei are counterstained with DAPI (blue). Scale bars: (a) and (c) 200 *μ*m; (b) and (d) 100 *μ*m; (e) and (f) 50 *μ*m. DAPI = 4′,6-diamidino-2-phenylindole; MSC = mesenchymal stem cell.

To test if OM-MSCs have the capacity to differentiate into fat cells, osteogenesis differentiation factors were added. After induction for 18 days, the OM-MSCs became shorter and bigger. Highly refractive vacuoles were observed under phase contrast microscope (Figure 4(a)). After Oil red O staining, a large number of lipid droplets in the cytoplasm were stained red (Figures 4(a) and 4(b)).

#### 3.4.2. *In Vitro* Osteogenic Assay

To test if OM-MSCs have the capacity to differentiate into bone cells, adipogenesis differentiation factors were added, too. After induction, OM-MSCs became cubical and showed tendency to aggregate. The alizarin red S was positively stained after osteogenic induction for 3 weeks, suggesting that the OM-MSCs have the potential to differentiate into osteoblasts* in vitro* (Figures 4(c) and 4(d)).

#### 3.4.3. *In Vitro* Neurogenic Assay

**Figure 5:**
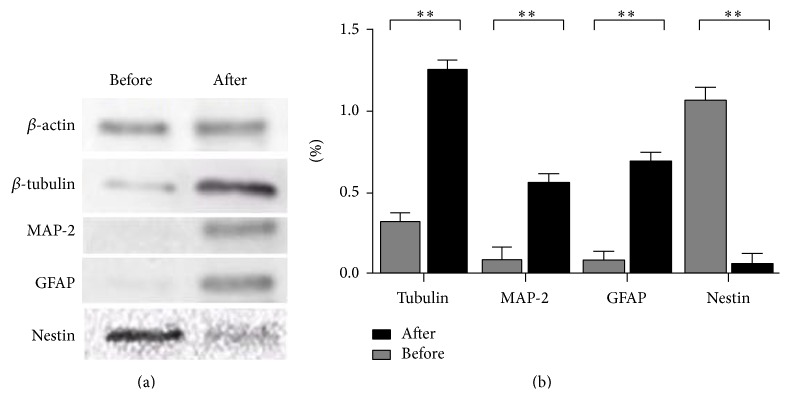
Western blot assay of OM-MSCs neuronal induction for 1 and 2 weeks. The differentiation of neuron-like cells from mesenchymal stem cells (MSCs) was verified by western blot. After induced, the neuron marker *β*-tubulin and MAP-2 were highly increased, and the stem cell marker, nestin, indicated significant downregulation.

To test if OM-MSCs have the capacity to differentiate into neuronal-like cells, cells were harvested and reseeded into differentiation conditions. After induction with retinoic acid for 12 days, the typical morphology of the neurites was presented and the neuron-like cell marker MAP-2 expression was revealed by immunofluorescence (Figures 4(e) and 4(f)). The following western blotting showed that OM-MSCs were expressing neuron-like cell marker MAP-2, *β*-TUB, and the glia marker GFAP. At the same time, the expression level of stem cell marker nestin was observably decreased (Figure 5).

### 3.5. Separation and Identification of Secreted Proteins

**Figure 6:**
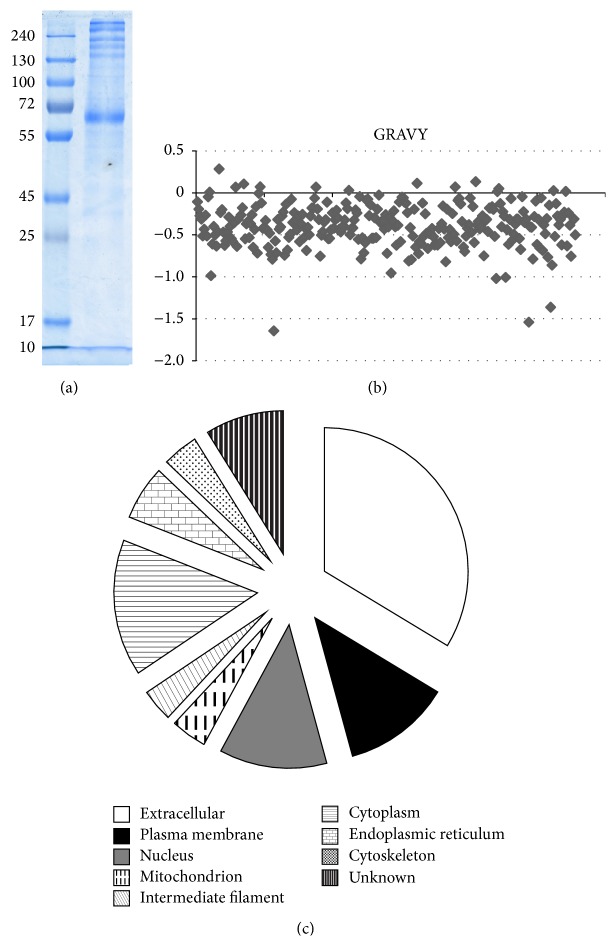
The SDS-PAGE analysis of the proteins isolated from OM-MSC conditioned medium and the hydrophobicity and primary subcellular localization of the identified proteins in OM-MSC conditioned medium. SDS-PAGE analysis of the secreted proteins of OM-MSC (a). The GRAVY values varied from −1.647 to 0.282 and 261 (94.5%) of them are negative, showing these proteins were hydrophilic and might be unidentified secreted proteins and functioned as growth factors or signaling molecules (b). Of the 276 identified proteins from OM-MSC conditioned medium, 175 (63.4%) were recognized as extracellular proteins through GO cellular component assignment. Classification by cellular localization is redundant since a protein can be classified in more than one compartment (c).

The secreted proteins of OM-MSCs were prefractionated via 1D-SDS-PAGE (Figure 6(a)). The gels were cut into 15 parts equally and trypsinized by in-gel digestion. Trypsinized peptides were analyzed by MS in triplicate and the results were processed using a sequential analysis as described in the proteomic data processing section. Shotgun proteomics with these rigorous conditions identified a total of 274 nonredundant proteins in OM-MSC conditioned medium. The entire list of identified proteins was provided in Table S1 (http://1000eb.com/1g25l) (see Supplementary Material available online at http://dx.doi.org/10.1155/2016/1243659).

The hydrophobicity property of proteins was expressed as the GRAVY index and the ProtParam tool at ExPASy was used to calculate the GRAVY values of proteins. The proteins exhibiting positive GRAVY values were recognized to be hydrophobic and those with negative values were deemed hydrophilic. As for the identified extracellular proteins in this study, the GRAVY values varied from −1.647 to 0.282 and 261 (94.5%) of them are negative (Figure 6(b)), showing that these proteins were hydrophilic.

Of the 276 identified proteins from OM-MSC conditioned medium, 175 (63.4%) were recognized as extracellular proteins through GO cellular component assignment (Figure 6(c)). Other identified proteins included plasma membrane (63, 22.8%), nucleus (63, 22.8%), mitochondrion (21, 7.6%), intermediate filament (19, 6.9%), cytoplasm (80, 29.0%), endoplasmic reticulum (32, 11.6%), cytoskeleton (21, 7.6%), and unknown (46, 16.7%). Classification by cellular localization is redundant since a protein can be classified into more than one compartment.

The molecular functions of the identified secreted proteins were composed of several categories, such as antioxidant activity, binding, catalytic activity, enzyme regulator activity, receptor activity, signal transducer activity, structural molecule activity, and transporter activity.

### 3.6. Biological Processes and Function Annotation

**Figure 7:**
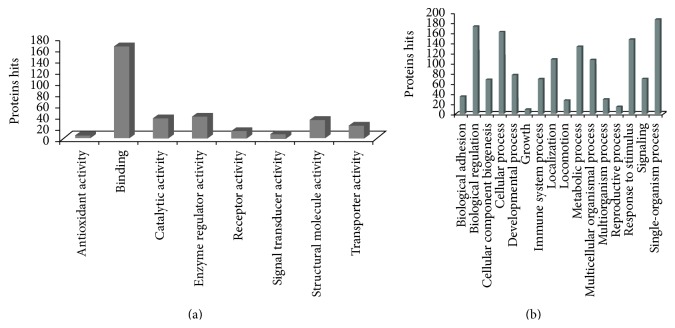
Molecular functions and biological processes of extracellular proteins identified in OM-MSC conditioned medium. The molecular functions of the identified secreted proteins were composed of several categories, such as antioxidant activity, binding, catalytic activity, enzyme regulator activity, receptor activity, signal transducer activity, structural molecule activity, and transporter activity (a). It indicated that the main functions of secreted proteins are maintaining the homeostasis of extracellular matrix, transmitting signals, and so on. Significantly higher frequencies of genes (*p* < 0.05) were associated with 16 biological processes (b). These proteins were most frequently involved in metabolism, defense response, signaling, and tissue differentiation.

**Figure 8:**
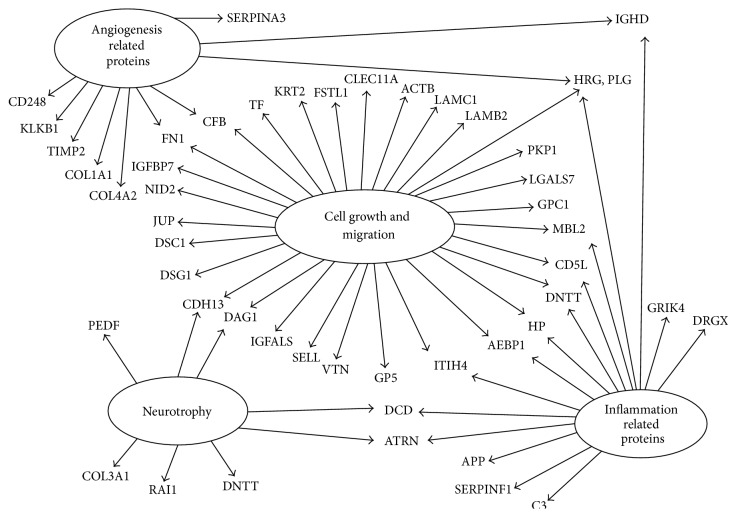
Function annotation of the identified secreted proteins. Proteins that have the function annotations in Swiss-Prot database were classified into 4 groups named angiogenesis related proteins, related to neurotrophy, related to cell growth, migration, differentiation, and apoptosis, and proteins associated with inflammation.

To investigate whether the secreted factors could function in the tissues repairment, GO analysis was conducted. Significantly higher frequencies of genes (*p* < 0.05) were associated with 16 biological processes (Figure 7). These proteins were most frequently involved in metabolism, defense response, signaling, and tissue differentiation. Proteins that have the function annotations in Swiss-Prot database were classified into 4 groups named related to neurotrophy, related to blood circulation, related to cell growth, differentiation, and apoptosis, and proteins associated with inflammation (Table 1, Figure 8).

## 4. Discussion

OM-MSCs have significantly clonogenic activity and could be easily propagated for the purpose of transplantation [16]. It indicated that these cells might be obtained from patients themselves and used for autologous transplantation [17, 19–22]. Previous studies have shown that OM-MSCs possessed multidirectional differentiation capacity [16]. As we confirmed here, OM-MSCs have the abilities to differentiate into mesoderm and ectoderm cell type, not only adipose cell or bone cells, but also neurons.

It has been agreed that the secretion of paracrine factors could contribute to the reparative effects of MSCs [25, 32, 33]. So far, the secretion proteome of several MSCs has been investigated [34–38]. However, the secretion proteome of OM-MSCs has not been investigated yet. In the present study we assessed the secretion proteome of MSCs by LC-MS. Our study identified 274 proteins in OM-MSCs conditioned medium and represents to date the first list of secretome of OM-MSCs. The current data showed that about 94.5% of identified proteins were hydrophilic and 63.4% of candidate proteins were located extracellularly. At the same time, it is still found that intracellular proteins from both the cytoplasmic and nuclear compartments accounted for 29% and 22% of candidate proteins. These intracellular proteins may be derived from the death cells.

To enhance our understanding of the paracrine effects of MSCs, the secreted proteins were classified according to their biological processes and molecular functions. Computational analysis predicted many processes that are generally associated with the functions of transplantation such as biological regulation, cellular process, development process, metabolic process, and response to stimulus. These processes would have reparative effects on most injured or diseased tissues, as their facilitation of immune cell migration to the site of injury, ECM remodeling, and an increase in the cellular metabolism [39].

It is believed that the promotion of OM-MSCs transplantation for treating CNS injuries is involved in the following four areas. (1) Cell growth and migration: the genes implicated in cell cycle were present in the OM-MSCs. Transferrin, IGF binding protein family members (IGFBP2, IGFBP3, IGFBP4, and IGFBP7), and SPARC are all reported to be involved in cell proliferation, migration, and differentiation. Plasminogen, microtubule-associated protein, clusterin, and collagen alpha-2 are known to be involved in cell apoptosis. (2) Angiogenesis and blood circulation: OM-MSCs expressed a number of genes implicated in regulation of blood circulation, that is, platelet glycoprotein V, carboxypeptidase N subunit 2, hemopexin, and even regulated angiogenesis, that is, plasminogen, histidine-rich glycoprotein, and pigment epithelium-derived factor (PEDF). (3) Inflammation/immune regulation: many of the genes are implicated in inflammation and immune regulations, that is, complement C3 and C4, CD5 antigen-like, and attractin. (4) Neurotrophy: OM-MSCs also expressed the essential genes that provide nutrition for nerve. Dystroglycan, dorsal root ganglia homeobox protein, and PEDF are known to play key roles in neural regulation, growth, and development. Thus we focused on proteins in these four areas. 45 proteins identified in this work were related in cell cycle like cell growth, development, differentiation, and apoptosis. 27 proteins were associated with the blood cycle, such as angiogenesis and blood coagulation. The immune regulation and neurotrophy related proteins were 28 and 9. Interestingly, there were plenty of proteins that are involved in more than one category especially some of which were tightly associated with neural growth and development suggested that OM-MSCs could be an effective source for repair of injured CNS and may play a critical role in OM-MSCs transplantation.

The IGF is a classical protein family regulating cell growth, survival, and differentiation, which not only is involved in the regulation of brain growth, development, and myelination, but also affects cognition and biochemistry in the adult brain [40, 41]. The IGFBPs have been proposed (1) to act as transport proteins in plasma, (2) to prolong the half-lives of the IGFs in circulation, (3) to determine the tissue- and cell-specific localization of IGF-I and IGF-II, and (4) to control the biological actions of IGF-I and IGF-II by modulating their interactions with their receptors [42]. One of the members is IGFBP-2, which plays a crucial role in regulating cell proliferation, driving invasion, and suppressing apoptosis [43]. Additionally, IGFBP-2 has been demonstrated to associate with the cell membranes of the rat olfactory bulb via proteoglycans in a RGD motif-independent manner [44]. Furthermore, OM-MSCs secrete other IGFs just like IGFBP-3, IGFBP-4, IGFBP-6, and IGFBP-7.

OM-MSCs also secreted molecules involved in neural differentiation and other functions. Dystroglycan can organize axon guidance cue location which is critical for nervous system development [45]. As an ECM, dystroglycan plays important roles in perisynaptic and axonal matrix formations and contributes to synaptic homeostatic plasticity [46]. The loss or decreased expression of dystroglycan could lead to poor prognosis in different malignant tumors [47–49] which suggested that dystroglycan may play the crucial roles during the repair of damage. Other proteins like dermcidin (DCD), retinoic acid induced 1 (RAI1), and cadherin 13 not only contribute to cell cycle related events, but also play roles in neural differentiation. DCD is a neural growth and survival factor and its therapeutic inhibition may be an effective treatment in a subset of breast carcinomas [50]. RAI1 is involved in neurobehavioral disorders [51] and plays a critical role in normal neural and craniofacial development [52]. Cadherin 13 is the member of a family of calcium-dependent cell-cell adhesion proteins and acts as a regulator of neural cell growth [53].

CD248 or endosialin was originally described as tumor endothelial marker which played a role in tumor angiogenesis [54–56]. CD248 is expressed not only in different kinds of tumors or cancer such as human carcinomas, sarcomas, and neuroectodermal tumors [57–59], but also during physiological processes including corpus luteum formation and wound healing [60]. Interestingly, recent studies reveal that the induction of CD248 expression by hypoxia is mediated by HIF-2*α* [61]. It turned out that CD248 is a marker for activated mesenchymal cells [62]. Therefore, we believe that CD248 could be developed as potential marker of OM-MSCs. Other CD molecules like CD5L may play a role in the regulation of the immune system [63].

Pigment-epithelium-derived factor (PEDF) belongs to serine protease inhibitor (serpin) superfamily that has been widely expressed among many tissues. Traditionally, PEDF is initially identified as a strong antiangiogenic factor [64], while recently increasing evidences demonstrate that PEDF is also a neurotrophic factor [65]. PEDF can improve neuronal survival and protect neurons including motor neurons [66], hippocampal neurons [67], dopaminergic midbrain neurons [65], and striatal neurons [68] in different toxin-induced models. Moreover PEDF has been linked with stem cell biology, and there are now accumulating researches showing PEDF support stem cell survival and maintaining multipotency of stem cells [69].

Besides, we found that the apolipoprotein E, insulin-like growth factor-binding protein 2 (IGF-BP2), clusterin, and other 38 proteins are not only detected in OM-MSC conditioned medium, but also detected in secretion proteome of OECs [70], indicating that there are some certain connections between them.

The paracrine hypothesis of MSCs avoids the immune compatibility, tumorigenicity, infections, and costs caused by the cell-based therapy, so that this will introduce a radically different dimension in regenerative medicine. Proteomics is an involved subject which focuses on large-scale study of proteins and their functions [71]. Through this method, we can discover truly effective components. Our results are valuable in the elucidation of the underlying mechanisms of OM-MSC. Such an approach will have a greater potential for the development of “off-the-shelf” MSC-based therapeutics.

## 5. Conclusion

Our findings suggested that OM-MSCs could secrete multiple trophic factors which might play important role in CNS regeneration. Although not fully understood, the role of OM-MSCs in neuronal regeneration and endogenous CNS repair functions is now largely acknowledged. Our ongoing study will focus on the roles of these molecules during development and regeneration of the primary olfactory system. It might improve our understanding about OM-MSCs and illuminate the application of OM-MSCs therapeutically.

## Supplementary Material

Proteins identified in three parallel tests by mass spectrum were shown in the supplementary table. As the result, a total of 274 nonredundant peoteins in OM-MSC conditioned medium were included in the table.

## Figures and Tables

**Table 1:** Function classification of OM-MSCs secreted proteins.

Cell growth/proliferation/differentiation/apoptosis/survival/adhesion/migration		
Accession	Gene name	Protein description
P02787	TF	Serotransferrin
Q14118	DAG1	Dystroglycan
Q8IUX7	AEBP1	Adipocyte enhancer-binding protein 1
P00751	CFB	Complement factor B
P35908	KRT2	Keratin, type II cytoskeletal 2 epidermal
Q12841	FSTL1	Follistatin-related protein 1
Q9Y240	CLEC11A	C-type lectin domain family 11 member A
P60709	ACTB	Actin, cytoplasmic 1
P11047	LAMC1	Laminin subunit gamma-1
P55268	LAMB2	Laminin subunit beta-2
P00738	HP	Haptoglobin
P04053	DNTT	DNA nucleotidylexotransferase
P14923	JUP	Junction plakoglobin
Q13835	PKP1	Plakophilin-1
Q14624	ITIH4	Inter-alpha-trypsin inhibitor heavy chain H4
O43866	CD5L	CD5 antigen-like
P11226	MBL2	Mannose-binding protein C
P04196	HRG	Histidine-rich glycoprotein
P17936	IGFBP3	Insulin-like growth factor-binding protein 3
P18065	IGFBP2	Insulin-like growth factor-binding protein 2
P22692	IGFBP4	Insulin-like growth factor-binding protein 4
P35052	GPC1	Glypican-1
P47929	LGALS7	Galectin-7 (Gal-7)
Q6E0U4	DMKN	Dermokine
Q7Z5J4	RAI1	Retinoic acid-induced protein 1
P00747	PLG	Plasminogen
Q71UI9	H2AFV	Histone H2A.V
Q66K74	MAP1S	Microtubule-associated protein 1S
P10909	CLU	Clusterin
Q9UBP9	GULP1	PTB domain-containing engulfment adapter protein 1
P55290	CDH13	Cadherin-13
P02751	FN1	Fibronectin
Q12913	PTPRJ	Receptor-type tyrosine-protein phosphatase eta
P40197	GP5	Platelet glycoprotein V
P04004	VTN	Vitronectin
P14151	SELL	L-selectin
P35858	IGFALS	Insulin-like growth factor-binding protein complex acid labile subunit
Q02413	DSG1	Desmoglein-1
Q08554	DSC1	Desmocollin-1
Q14112	NID2	Nidogen-2
Q16270	IGFBP7	Insulin-like growth factor-binding protein 7
Q86SJ6	DSG4	Desmoglein-4
Q9UI47	CTNNA3	Catenin alpha-3
Q9Y6C2	EMILIN1	EMILIN-1
F5GY03	SPARC	SPARC

Angiogenesis and blood circulation		
P00751	CFB	Complement factor B
P04196	HRG	Histidine-rich glycoprotein
P00747	PLG	Plasminogen
P00734	F2	Prothrombin
P05154	SERPINA5	Plasma serine protease inhibitor
P02763	ORM1	Alpha-1-acid glycoprotein 1
P02774	GC	Vitamin D-binding protein
P00748	F12	Coagulation factor XII
P01008	SERPINC1	Antithrombin-III
P01009	SERPINA1	Alpha-1-antitrypsin
P01019	AGT	Angiotensinogen
P01042	KNG1	Kininogen-1
P02749	APOH	Beta-2-glycoprotein 1
P02768	ALB	Serum albumin
P05155	SERPING1	Plasma protease C1 inhibitor
P07359	GP1BA	Platelet glycoprotein Ib alpha chain
P12259	F5	Coagulation factor V
P68871	HBB	Hemoglobin subunit beta
Q9UNN8	PROCR	Endothelial protein C receptor
P02649	APOE	Apolipoprotein E
P08572	COL4A2	Collagen alpha-2(IV) chain
P01011	SERPINA3	Alpha-1-antichymotrypsin
P02452	COL1A1	Collagen alpha-1(I) chain
P03952	KLKB1	Plasma kallikrein
P16035	TIMP2	Metalloproteinase inhibitor 2
Q9HCU0	CD248	Endosialin
P02751	FN1	Fibronectin

Inflammation/immune regulation		
Q8IUX7	AEBP1	Adipocyte enhancer-binding protein 1
P00738	HP	Haptoglobin
P04053	DNTT	DNA nucleotidylexotransferase
P81605	DCD	Dermcidin
P01024	C3	Complement C3
P01880	IGHD	Ig delta chain C region
O43866	CD5L	CD5 antigen-like
P11226	MBL2	Mannose-binding protein C
P04196	HRG	Histidine-rich glycoprotein
P01871	IGHM	Ig mu chain C region
P01876	IGHA1	Ig alpha-1 chain C region
P01877	IGHA2	Ig alpha-2 chain C region
P05546	SERPIND1	Heparin cofactor 2
P07357	C8A	Complement component C8 alpha chain
P0C0L4	C4A	Complement C4-A
P10643	C7	Complement component C7
P13671	C6	Complement component C6
P15814	IGLL1	Immunoglobulin lambda-like polypeptide 1
P19652	ORM2	Alpha-1-acid glycoprotein 2
P48740	MASP1	Mannan-binding lectin serine protease 1
Q6UXS9	CASP12	Inactive caspase-12
P00747	PLG	Plasminogen
P00734	F2	Prothrombin
P05154	SERPINA5	Plasma serine protease inhibitor
P02763	ORM1	Alpha-1-acid glycoprotein 1
P02774	GC	Vitamin D-binding protein

Neural regulation/growth/development		
Q14118	DAG1	Dystroglycan
Q16099	GRIK4	Glutamate receptor ionotropic, kainate 4
P05067	APP	Amyloid beta A4 protein
A6NNA5	DRGX	Dorsal root ganglia homeobox protein
P36955	SERPINF1	Pigment epithelium-derived factor
P04053	DNTT	DNA nucleotidylexotransferase
P81605	DCD	Dermcidin
Q7Z5J4	RAI1	Retinoic acid-induced protein 1
P55290	CDH13	Cadherin-13
